# Supplementary material for: An airway-to-brain sensory pathway mediates influenza-induced sickness
Source: Nature. 2023 Mar 8;615(7953):660–7. doi: 10.1038/s41586-023-05796-0 (PMC10033449; doi:10.1038/s41586-023-05796-0)
Supplement: Supplementary file 1 — Reporting Summary [file 41586_2023_5796_MOESM1_ESM.pdf]

nature portfolio

Corresponding author(s): Stephen D. Liberles

Last updated by author(s): Jan 12, 2023

Reporting Summary

Nature Portfolio wishes to improve the reproducibility of the work that we publish. This form provides structure for consistency and transparency in reporting. For further information on Nature Portfolio policies, see our Editorial Policies and the Editorial Policy Checklist.

Statistics

For all statistical analyses, confirm that the following items are present in the figure legend, table legend, main text, or Methods section.

n/a	Confirmed	
☐	☒	The exact sample size (*n*) for each experimental group/condition, given as a discrete number and unit of measurement
☐	☒	A statement on whether measurements were taken from distinct samples or whether the same sample was measured repeatedly
☐	☒	The statistical test(s) used AND whether they are one- or two-sided<br>*Only common tests should be described solely by name; describe more complex techniques in the Methods section.*
☒	☐	A description of all covariates tested
☐	☒	A description of any assumptions or corrections, such as tests of normality and adjustment for multiple comparisons
☐	☒	A full description of the statistical parameters including central tendency (e.g. means) or other basic estimates (e.g. regression coefficient) AND variation (e.g. standard deviation) or associated estimates of uncertainty (e.g. confidence intervals)
☐	☒	For null hypothesis testing, the test statistic (e.g. *F*, *t*, *r*) with confidence intervals, effect sizes, degrees of freedom and *P* value noted<br>*Give P values as exact values whenever suitable.*
☒	☐	For Bayesian analysis, information on the choice of priors and Markov chain Monte Carlo settings
☒	☐	For hierarchical and complex designs, identification of the appropriate level for tests and full reporting of outcomes
☒	☐	Estimates of effect sizes (e.g. Cohen's *d*, Pearson's *r*), indicating how they were calculated

Our web collection on statistics for biologists contains articles on many of the points above.

Software and code

Policy information about availability of computer code

Data collection	Leica Confocal LAS AF for confocal images. Zeiss ZEN for alkaline phosphatase images. Logitech Capture (2.08.11) for motility recordings. Synapses software (Build: 94-42329P, Tucker-Davis Technology) for fiber photometry recordings. iox software (2.10.8, Emka Technologies) for telemetry based body temperature recordings.
Data analysis	ImageJ (1.53q), R Studio (4.1.2), Seurat (4.1.0), Matlab (R2020a), GraphPad Prism (8.4.3), and SPSS (21) were used for for data analysis.

For manuscripts utilizing custom algorithms or software that are central to the research but not yet described in published literature, software must be made available to editors and reviewers. We strongly encourage code deposition in a community repository (e.g. GitHub). See the Nature Portfolio guidelines for submitting code & software for further information.

Data

Policy information about availability of data

All manuscripts must include a data availability statement. This statement should provide the following information, where applicable:

- Accession codes, unique identifiers, or web links for publicly available datasets
- A description of any restrictions on data availability
- For clinical datasets or third party data, please ensure that the statement adheres to our policy

All data used to generate figures, including behavioral data points from each individual animal, are provided as source data. Gene expression analysis involved previously published and already fully available single cell RNA sequencing data, and the accession number for that data (GEO:GSE145216) is now reported in this paper.

Field-specific reporting

Please select the one below that is the best fit for your research. If you are not sure, read the appropriate sections before making your selection.

☒ Life sciences ☐ Behavioural & social sciences ☐ Ecological, evolutionary & environmental sciences

For a reference copy of the document with all sections, see nature.com/documents/nr-reporting-summary-flat.pdf

Life sciences study design

All studies must disclose on these points even when the disclosure is negative.

Sample size	Sample sizes were chosen based on prior expertise and publications in our field (for example, Baral et al., Nature Medicine, 2018, Ilanges et al., Nature, 2022) and are disclosed in each figure legend.
Data exclusions	No data were excluded from the analysis.
Replication	All experiments where representative images were depicted were independently replicated at least twice, and typically three times, as detailed for each experiment in figure legends.
Randomization	Animals were randomly assigned to experimental cohorts, based only genotype and appropriate age-matching.
Blinding	The same investigator performed genotyping and analysis of sickness responses, so data were not generated blind to genotype or experimental group. Nonetheless, all animals in each experiment were analyzed without bias.

Reporting for specific materials, systems and methods

We require information from authors about some types of materials, experimental systems and methods used in many studies. Here, indicate whether each material, system or method listed is relevant to your study. If you are not sure if a list item applies to your research, read the appropriate section before selecting a response.

Materials & experimental systems

n/a	Involved in the study	
☐	☒	Antibodies
☒	☐	Eukaryotic cell lines
☒	☐	Palaeontology and archaeology
☐	☒	Animals and other organisms
☒	☐	Human research participants
☒	☐	Clinical data
☒	☐	Dual use research of concern

Methods

n/a	Involved in the study	
☒	☐	ChIP-seq
☒	☐	Flow cytometry
☒	☐	MRI-based neuroimaging

Antibodies

Antibodies used	Chicken anti-GFP primary antibody- Aves Labs, Cat number: GFP-1020, The Antibody Registry ID: AB_10000024.<br>Rabbit anti-RFP primary antibody- Rockland, Cat number: 600-401-379, The Antibody Registry ID: AB_2209751.<br>Goat anti-DTR primary antibody- R&D Systems, Cat number: AF-259-NA, The Antibody Registry ID: AB_354429.<br>Rabbit anti-COX2 primary antibody- Abcam, Cat number: ab179800, The Antibody Registry ID: AB_2894871.<br>Anti-goat Alexa488 secondary antibody- Jackson Immunoresearch, Cat number: 705-545-147.<br>Anti-rabbit Alexa488 secondary antibody- Jackson Immunoresearch, Cat number: 711-545-152.<br>Anti-rabbit Cy3 secondary antibody- Jackson Immunoresearch, Cat number: 111-165-144.<br>Anti-chicken Alexa647 secondary antibody- Jackson Immunoresearch, Cat number: 703-605-155.
Validation	Primary antibodies (rabbit anti-COX2 antibody, goat anti-DTR antibody, chicken anti-GFP antibody, rabbit anti-RFP antibody are commercially available, extensively used in prior studies,and for anti-COX2 validated in knockout animals by the manufacturer. In our previous work with anti-DTR, GFP, and RFP antibodies, background staining was not observed in wild type animals lacking antigen.

Animals and other organisms

Policy information about studies involving animals; ARRIVE guidelines recommended for reporting animal research

Laboratory animals	Animals were maintained under constant temperature (23 ± 1°C) and relative humidity (46 ± 5%) with a 12-h light/dark cycle. Wild-type C57BL/6J (000664), Nestin-Cre (003771), Phox2b-Cre (016223), Advillin-CreER (032027), Trpv1-Cre (017769), Pdyn-ires-Cre (027958), Piezo2-ires-Cre (027719), Oxtr-ires-Cre (031303), Agrp-Cre (012899), lsl-Dtr (007900), lsl-tdTomato (Ai14, 007914) were

	purchased from Jackson Laboratories. Gabra1-ires-Cre, lsl-L10-Gfp, and flox-Ptger3 mice were previously generated (citations provided in text). There was no sex bias and the age of the animals was 6-8 weeks.
Wild animals	No wild animals were used.
Field-collected samples	No field-collected samples were used.
Ethics oversight	All animal procedures followed the ethical guidelines outlined in the NIH Guide for the Care and Use of Laboratory Animals, and all protocols were approved by the institutional animal care and use committee (IACUC) at Harvard Medical School.

Note that full information on the approval of the study protocol must also be provided in the manuscript.
